# Ultra-Low Power Sensor Devices for Monitoring Physical Activity and Respiratory Frequency in Farmed Fish

**DOI:** 10.3389/fphys.2019.00667

**Published:** 2019-05-29

**Authors:** Juan Antonio Martos-Sitcha, Javier Sosa, Dailos Ramos-Valido, Francisco Javier Bravo, Cristina Carmona-Duarte, Henrique Leonel Gomes, Josep Àlvar Calduch-Giner, Enric Cabruja, Aurelio Vega, Miguel Ángel Ferrer, Manuel Lozano, Juan Antonio Montiel-Nelson, Juan Manuel Afonso, Jaume Pérez-Sánchez

**Affiliations:** ^1^Nutrigenomics and Fish Growth Endocrinology Group, Institute of Aquaculture Torre de la Sal, Consejo Superior de Investigaciones Científicas (CSIC), Castellón, Spain; ^2^Department of Biology, Faculty of Marine and Environmental Sciences, Instituto Universitario de Investigación Marina (INMAR), Campus de Excelencia Internacional del Mar (CEI-MAR), University of Cádiz, Cádiz, Spain; ^3^Institute for Applied Microelectronics (IUMA), University of Las Palmas de Gran Canaria, Las Palmas, Spain; ^4^Institute of Microelectronics of Barcelona (IMB-CNM), Consejo Superior de Investigaciones Científicas (CSIC), Barcelona, Spain; ^5^Technological Centre for Innovation in Communications (iDeTIC), University of Las Palmas de Gran Canaria, Las Palmas, Spain; ^6^Centre for Marine Sciences (CCMAR), Universidade do Algarve, Faro, Portugal; ^7^Aquaculture Research Group, Institute of Sustainable Aquaculture and Marine Ecosystems (IU-ECOAQUA), University of Las Palmas de Gran Canaria, Las Palmas, Spain

**Keywords:** aquaculture, sensor, swimming tests, fish welfare, physical activity, respiratory frequency, oxygen consumption

## Abstract

Integration of technological solutions aims to improve accuracy, precision and repeatability in farming operations, and biosensor devices are increasingly used for understanding basic biology during livestock production. The aim of this study was to design and validate a miniaturized tri-axial accelerometer for non-invasive monitoring of farmed fish with re-programmable schedule protocols. The current device (AE-FishBIT v.1s) is a small (14 mm × 7 mm × 7 mm), stand-alone system with a total mass of 600 mg, which allows monitoring animals from 30 to 35 g onwards. The device was attached to the operculum of gilthead sea bream (*Sparus aurata*) and European sea bass (*Dicentrarchus labrax*) juveniles for monitoring their physical activity by measurements of movement accelerations in *x*- and *y*-axes, while records of operculum beats (*z*-axis) served as a measurement of respiratory frequency. Data post-processing of exercised fish in swimming test chambers revealed an exponential increase of fish accelerations with the increase of fish speed from 1 body-length to 4 body-lengths per second, while a close relationship between oxygen consumption (MO_2_) and opercular frequency was consistently found. Preliminary tests in free-swimming fish kept in rearing tanks also showed that device data recording was able to detect changes in daily fish activity. The usefulness of low computational load for data pre-processing with on-board algorithms was verified from low to submaximal exercise, increasing this procedure the autonomy of the system up to 6 h of data recording with different programmable schedules. Visual observations regarding tissue damage, feeding behavior and circulating levels of stress markers (cortisol, glucose, and lactate) did not reveal at short term a negative impact of device tagging. Reduced plasma levels of triglycerides revealed a transient inhibition of feed intake in small fish (sea bream 50–90 g, sea bass 100–200 g), but this disturbance was not detected in larger fish. All this considered together is the proof of concept that miniaturized devices are suitable for non-invasive and reliable metabolic phenotyping of farmed fish to improve their overall performance and welfare. Further work is underway for improving the attachment procedure and the full device packaging.

## Introduction

Biosensor technology is increasingly used for a non-invasive measurement during farming and experimental studies of a range of variables that are directly or indirectly relevant for animal health and welfare (Ivanov et al., 2015; Neethirajan et al., 2017). Thus, cameras, sonars, acoustic telemetry and environmental biosensors of chemical and biological safety risk factors are currently used to improve feed conversion, product quality and disease prevention, reducing at the same time the environmental impact of farming operations (Saberioon et al., 2017; Føre et al., 2018; Enviguard EU project^1^). Such technological solutions contribute to the estimation of fish biomass for ration size assignment and medicinal dosages, or to the design of automated feeding strategies based on fish behavior in order to avoid unnecessary feed waste. Furthermore, the concept of sentinel animals fitted with biosensors, initially developed for the dairy industry (Simbeye et al., 2014), has been extended to the culture of oysters (Andrewartha et al., 2016) and fish (Staaks et al., 2010) with abundant literature on measurements of body temperature, animal position and depth in both wild and farmed fish (e.g., Clark et al., 2008; Payne et al., 2014; Metcalfe et al., 2016, among others). This approach is essential for the optimization of aquaculture operations, and the assessment of welfare and stress in farmed fish becomes a major challenge for a more efficient and ethical animal production.

With the advent of the MEMS (MicroElectroMechanical System) technology, high-precision and low-cost accelerometers are available as components of different electronic gadgets of physical activity (activity trackers, fitness band and heart rate monitors, sport-watches, smart pedometers and wellness monitors, among others). Acceleration data-loggers alone or in combination with pressure, temperature and heart-rate biosensors have also been used for tracing movement and estimating activity-specific energy expenditure or feeding behavior in a number of fish species, including juvenile hammerhead sharks (*Sphyrna lewini*, Gleiss et al., 2010), sockeye salmon (*Oncorhynchus nerka*, Clark et al., 2010), European sea bass (*Dicentrarchus labrax*, Wright et al., 2014), Atlantic cod (*Gadus morhua*, Claireaux et al., 1995), Atlantic salmon (*Salmo salar*, Kolarevic et al., 2016) and red-spotted groupers (*Epinephelus akaara*, Horie et al., 2017). These sensors operate as acoustic transmitter tags that contain a tri-axial accelerometer, which registers gravity forces and acceleration in the *x*-, *y*-, and *z*-directions. Currently, the smallest market size for these tags is 7 mm diameter and 20 mm length with a weight of 2.6 g in air and a typical battery life of 1–3 months, depending on measurement periods and transmission intervals. However, as pointed out by Kolarevic et al. (2016), the interference between transmitted signals limits the use of a large number of acceleration tags in a single rearing unit or in two neighboring units. Otherwise, in the aquatic environment, the use of low radiofrequency transmission is limited to 1–2 m of maximum communication range without repeaters (Palmeiro et al., 2011). Thus, with the double aim to produce small sensors and to combine measurements of physical activity and energy demand, the design and production of ultra-low power stand-alone devices using available technology was conducted within the AQUAEXCEL^2020^ EU project. The resulting device (AE-FishBIT) was designed to be implanted on fish operculum for measurements of physical activity and respiratory frequency. To the best of our knowledge, there is not in the market any device designed to provide simultaneously these two types of measurements. The present work was envisaged as the proof of concept that data acquired with the constructed prototype (AE-FishBIT v.1s) can be used in a reliable manner at different tank scales for the assessment of physical activity and energy expenditure status of farmed fish. This functional validation of the device has been conducted in European sea bass and gilthead sea bream (*Sparus aurata*) as the two most significant farmed fish of the Mediterranean aquaculture (FAO, 2018).

## Materials and Methods

### Hardware Architecture

**FIGURE 1:**
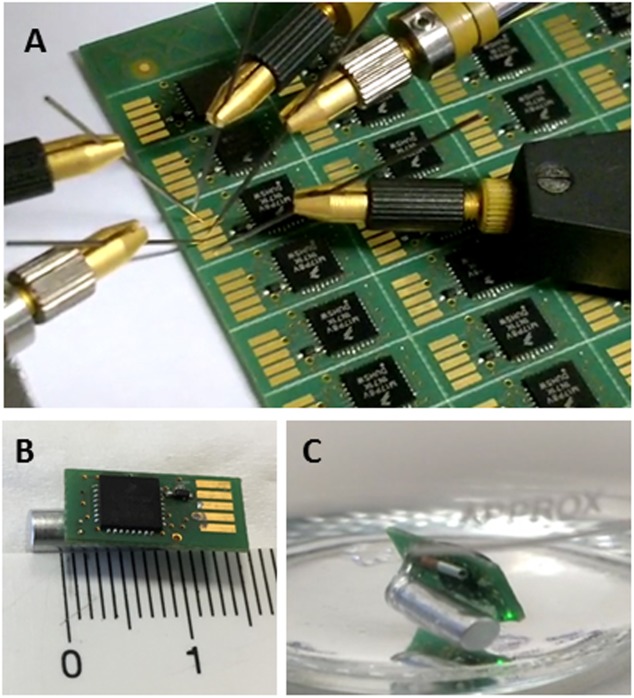
**(A)** Electrical probing of pcb-mounted devices. **(B)** Photograph of AE-FishBIT v.1s before encapsulation. **(C)** Encapsulated AE-FishBIT v.1s in underwater operation. Flashing led light indicates the end of data acquisition program for user reference.

The proposed device is a programmable and reconfigurable tri-axial accelerometer for measuring accelerations in *x*-, *y*-, and *z*-axes in the range of ±8 *g* and sampling frequencies up to 800 Hz. The basic components disposed on a 0.8 mm 4-layer printed circuit board (pcb) are: (i) one accelerometer (MMA8451Q; NXP Semiconductors Research, Eindhoven, Netherlands), (ii) one high performance Li-ion battery (UMAC040130A003TA01; muRata Electronics, Kyoto, Japan) of low charge time (120 s), (iii) one microprocessor (KL17; NXP Semiconductors Research) with 256 kb flash memory and 32 kb RAM memory and (iv) one RFID tagging device (nano-transponder ID-100A-1.25; TROVAN, Madrid, Spain) for rapid identification. System integration includes water-proof packaging (tested up to 6 bars of pressure in air and seawater environments) with isolated connector pins. The complete package size of the AE-FishBIT v.1s measures 14 mm × 7.2 mm × 7.0 mm with a total mass of 600 mg in air (Figure 1).

### Sensor Location and Attachment Procedure

**FIGURE 2:**
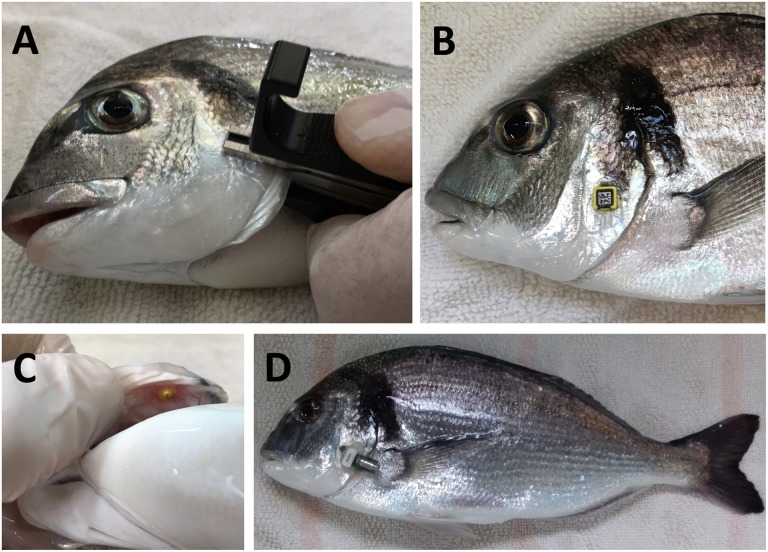
Device attachment to sea bream operculum. **(A)** RapID tag is attached to fish operculum with the tag piercing tool. The external side of the tag **(B)** allows fixation with adhesive of a 3D-printed pocket. **(C)** Internal side of attached sea bream operculum. **(D)** Sea bream with an attachment with the RapiID tag and 3D pocket procedure.

The operculum was chosen as the target location for the sensor as it allowed monitoring physical activity by measurements of accelerations in *x*- and *y*-axes, while records of operculum breathing (*z*-axis) served as a direct measurement of respiratory frequency. The device attachment was accomplished using small and light laboratory tags for identification of experimentation animals (RapID tags from RapID Lab, Inc., San Francisco, CA, United States) that were rapidly pierced to opercula of anesthetized fish (100 μg/mL 3-aminobenzoic acid ethyl ester MS-222, Sigma, St. Louis, MO, United States). In a second step, a rigid polyamide PA 2200 3D-printed pocket was fixed with an innocuous and quick drying aquarium adhesive (cyanoacrylate) to the exterior side of the RapID tag. Such approach allowed easy application and removal of the device in the pocket (Figure 2).

### Data Acquisition and Software Processing

Software data processing aimed to assess respiratory frequency and fish activity. The first estimation was carried out with the signal of the *z*-axis of the accelerometer, and the second one was derived from the *x*- and *y*-axis signals of the accelerometer.

The recorded *z*-axis accelerations generated positive and negative signals, related to operculum opening and closing, respectively. Other movements superimposed to this *z*-axis signal were angular accelerations that informed of fish trajectory or side head movement, though the dominant periodic component was always operculum movement. To calculate the respiratory frequency, the *z*-axis signal of the accelerometer was band pass filtered between 0.5 and 8 Hz to reduce the noise (measurements were expected below 5 Hz), highlighting the periodic properties of the signal. The numbers of maxima or minima in the recorded signals were registered at a sampling rate of f_s_ = 100 samples per second. Then, the signal was derived and the number of crosses through zero was divided by two to obtain the signal period.

The signal of the accelerometer was noisy, and the low pass filter was not able to eliminate some spurious crosses through zero. To alleviate this noise drawback and to obtain a more accurate estimation of the number of peaks, they were estimated in *N* consecutive frames of *T* seconds. Experimentally, it was calculated that around half of the frames included spurious crosses through zero, increasing the value of the respiratory frequency. As trade-off between complex signal denoising processing algorithms and the limited energy and computational capability of the microprocessor, the final number of peaks (*N_p_*) was defined as the 25% percentile of the *N* estimations. This 25% percentile was a conservative value below the 50% of usually noisy frames. Anyway, values between 20 and 40% provided similar results. Hence, the respiratory frequency was issued every *N* ⋅ *T* seconds and it was calculated as:

$$F_{resp}=N_{\text{p}}/T$$

Values of *N*, *T* and *f_s_* were heuristically chosen to assure a good statistical representation. The value of *f_s_* was established as 100 Hz. A value of T_s_ = 10 seconds was chosen as frame length. This value implied 5 peaks when the respiratory frequency was 2 Hz. *N* was established equal to 12 (respiratory frequency was calculated every 120 s), which allowed a reasonable estimation of the 25% percentile.

To describe the physical activity index of the fish spatio-temporal movement along the *x*- and *y*-axes, we relied on the simple optimization principle that the human movements strives to achieve maximally smooth movements by minimizing the first temporal derivative of acceleration, or “jerk,” while constraining all higher derivatives to zero (Flash and Hogan, 1985). This fact has been also applied to measure the intensity of the movement of terrestrial livestock (Hamäläinen et al., 2011). In this way, the energy of the jerk can be seen as a measure of the intensity of fish movement. To ease microprocessor calculations, the physical activity index was defined as the averaged energy of the jerk along the frame. Similarly to the respiratory rate, the energy of the jerk was estimated on *N* consecutive frames of *T* seconds and the 25% percentile provided the activity index measurement. The values of *N*, *T*, and *f_s_* were the same than in the respiratory frequency algorithm.

The procedure was as follows. The accelerations in the *x*- and *y*-axis, named a_x_(n) and a_y_(n), respectively, were derived as:

$$\begin{array}{l} d_{x}(n)=a_{x}(n)-a_{x}(n-1)\\ d_{y}(n)=a_{y}(n)-a_{y}(n-1) \end{array}$$

The standard deviations of *d_x_*(*n*) and *d_y_*(*n*) were calculated:

$$\sigma_{x}=\sqrt{\frac{\sum_{n=1}^{T\cdot f_{s}}{\left(d_{x}(n)-\mu_{x}\right)}^{2}}{T\cdot f_{s}}and\sigma_{y}}=\sqrt{\frac{\sum_{i=1}^{T\cdot f_{s}}{\left(d_{y}(n)-\mu_{y}\right)}^{2}}{T\cdot f_{s}}}$$

where μ_*x*_ and μ_*y*_ were the average of *d_x_*(*n*) and *d_y_*(*n*), respectively. The energy of the jerk was then obtained as:

$$E_{jerk}=\sqrt{\sigma_{x}^{2}(n)+\sigma_{y}^{2}(n)}$$

**FIGURE 3:**
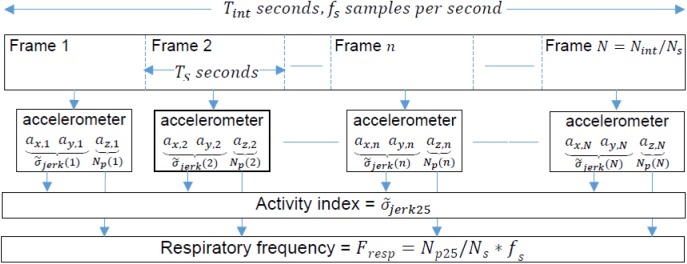
Procedure diagram for the calculation of respiratory frequency and physical activity index.

The physical activity index was the 25% percentile of the energies of the jerk estimated on *N* consecutive frames. A summary of the procedure for the calculation of respiratory frequency and physical activity index is shown in Figure 3.

Processing of the raw data generated by the tri-axial accelerometer and stored in the memory of the device required data transfer in a computer after tests. Using this procedure, the autonomy of the device was limited to the amount of memory, which was able to store a total of 6 min of raw data measured at a rate of 100 samplings per second. It was established that 2 min was a reliable time window for statistical calculations, and the device was then programmed to acquire three sets of 2 min of raw data for a given experimental schedule.

Software development also offered the possibility to process on-board recorded data by means of mathematical approximations to ease microprocessor function. This approach is not a high memory consuming process, and the autonomy of the system could be consequently increased up to 6 h of continuous data recording, allowing longer schedules as long as the battery of the device is operative. Algorithms for direct on-board calculation of respiratory frequency and physical activity index by the sensor were programmed through the FRMD-KL25Z algorithm platform and uploaded to the device. This step involved some changes on the algorithm to estimate the physical activity index due to the limited computational power of the sensor. For this purpose, the standard deviations of *d_x_*(*n*) and *d_y_*(*n*) were approximated by:

$$\sigma_{x}=\frac{\sum_{n=1}^{T\cdot f_{s}}\left|d_{x}(n)-\mu_{x}\right|}{T\cdot f_{s}}\text{ and }\sigma_{y}=\frac{\sum_{n=1}^{T\cdot f_{s}}\left|d_{y}(n)-\mu_{y}\right|}{T\cdot f_{s}}$$

The energy of the jerk was estimated as:

$$E_{jerk}=\sigma_{x}+\sigma_{y}.$$

The value of *T* was changed from 10 to 10.24 s. It means a frame of 1024 data, which is a power of 2, more convenient for the sensor programming.

### Functional Testing

Initial tests of the device were conducted in overnight fasted sea bream and sea bass juveniles (30–150 g body weight) reared in the indoor experimental facilities of Institute of Aquaculture Torre de la Sal (IATS-CSIC, Castellón, Spain) under natural photoperiod and temperature conditions (40°5′N; 0°10′E). Fish with the implanted device were exercised in an intermittent-closed swim tunnel respirometer of 10 L water volume (Loligo^®^ Systems, Viborg, Denmark). The swim tunnel was submerged into a water bath that served as a water reservoir for flushing the respirometer after each closed respirometer run (flush pump: Eheim 1048, 10 L/min; Deizisau, Germany). To ensure constant high water quality, the water bath was connected by a second flush pump (Eheim 1250, 20 L/min; Deizisau, Germany) to a 100 L reservoir tank coupled to a re-circulatory system equipped with physical and biological filters and a programmable temperature system fixed at 24–25°C. Sea water (80–95% O_2_ saturation) flowed back into the re-circulatory system by means of gravity, remaining unionized ammonia, nitrites and nitrates almost undetectable along the whole experiment. A thruster within the respirometer was used to generate a swimming current. Water velocities were calibrated with a hand-held digital flow meter that was ordered by the controller Movitrac^®^ LTE 0.37kW/0.5HP (SEW Eurodrive, Normanton, United Kingdom). Respirometry runs and chamber flushing were automatically controlled with the DAQ-M instrument (Loligo^®^ Systems) connected to a PC equipped with AutoResp^TM^ software (Loligo^®^ Systems). Water temperature and O_2_ saturation within the respirometer were measured using a Witrox 1 single channel O_2_ meter (Loligo^®^ Systems, Viborg, Denmark), equipped with a needle-type fiber optical micro-sensor (NTH, PreSens Precision Sensing GmbH, Regensburg, Germany) and a temperature probe suspended into the water current within the respirometer.

For testing procedures, anesthetized fish with the AE-FishBIT v.1s prototype were transferred into the swim tunnel and allowed to recover and acclimate at a swimming speed of 0.5–1.0 body-lengths per second (BL/s), until their measurements of O_2_ consumption rates (MO_2_) reached a constant low plateau. This was achieved when the regression line between the O_2_ consumption and time after transfer was not significantly different from zero during four consecutive 5 min intervals, and it typically happened after 30–45 min with MO_2_ around 220–240 mgO_2_/kg/h (Martos-Sitcha et al., 2018). After this acclimation period, water velocity was increased in 0.5 BL/s steps, and specimens were submitted to controlled speeds between 1 and 4 BL/s during the first testing approaches, or from 1 BL/s until exhaustion in the final on-board validation of data recording. The range between 1 and 4 BL/s for the first tests was chosen to assure that AE-FishBIT measurements were obtained in the linear increase of metabolic rates according to O_2_ consumption (Martos-Sitcha et al., 2018). Each swimming interval at a given velocity lasted 5 min, consisting in “flush-wait-measurement” cycles (60 s flush interval to exchange the respirometer water = “flush”; 30 s mixing phase in closed mode = “wait”; and a 210 s MO_2_ measuring period in closed mode = “measurement”). During the measurement interval, O_2_ saturation of the swim tunnel water was recorded every second. MO_2_ was automatically calculated by the AutoResp^TM^ software from linear decreases (*r*^2^ = 0.98–1.0) in chamber O_2_ saturation during the measurement period at each discrete and specific speed using the appropriate constants for O_2_ solubility in seawater (salinity, temperature, and barometric pressure). After the swim tunnel test, fish were anesthetized again to recover the AE-FishBIT for data download. For each fish, three data sets of 2 min raw data were acquired by the device at different speed steps. Additional tests were conducted in order to check the correlation of raw data calculated parameters with those coming from the on-board algorithms. To achieve this validation, devices were programmed to simultaneously store raw data (3 steps of 2 min data sets) and algorithm processed results.

For tests conducted with free-swimming fish (sea bream 292.5 ± 20.5 body weight, sea bass 161.6 ± 14.9 body weight) in 500 L tanks (stocking density ranging between 6 and 8 kg m^-3^ for both species), six devices were programmed to acquire 2 min raw data sets at three discrete times 1 day after device implantation (10:00 a.m., 2:00 p.m., and 6:00 p.m.). This schedule covered a wide range of possible circadian variations in activity and respiration indexes. Fish remained fasted during the day of data recording to avoid the possible distortion produced by feeding.

To check how blood markers of stress and welfare were affected by prototype implantation, polyamide PA 2200 dummy devices (same size and weight than the functional prototype) were implanted in active feeding sea bass (100–200 g) and sea bream of two different class of size (50–90 g; 300–500 g), reared at 22–24°C. After 1 week, tagged and non-tagged fish (*n* = 5–7 fish per group) were anesthetized and blood was quickly taken from caudal vessels with heparinized syringes. A blood aliquot was centrifuged at 3,000 ×*g* for 20 min at 4°C, and the plasma was stored at -80°C until the subsequent biochemical assays. Plasma glucose levels were measured by the glucose oxidase method (Thermo Fisher Scientific, Waltham, MA, United States) adapted to 96-well microplates. Blood lactate was measured in deproteinized samples (perchloric acid 8%) using an enzymatic method based on the use of lactate dehydrogenase (Instruchemie, Delfzijl, Netherlands). Plasma triglycerides (TG) were determined using lipase/glycerol kinase/glycerol-3-phosphate oxidase reagent (Thermo Fisher Scientific). Plasma cortisol levels were analyzed using an EIA kit (Kit RE52061 m IBL, International GmbH, Germany). The limit of detection of the assay was 2.46 ng/mL with intra- and inter-assay coefficients of variation lower that 3 and 5%, respectively.

### Ethics Statement

No mortalities were observed during fish manipulation and data recording, and all the described procedures were approved by the Ethics and Animal Welfare Committee of Institute of Aquaculture Torre de la Sal and carried out according to the National (Royal Decree RD53/2013) and the current EU legislation (2010/63/EU) on the handling of experimental fish.

### Statistical Analysis

Daily variation of respiratory frequency and physical activity index in free-swimming sea bream and sea bass was analyzed by one-way ANOVA. Blood parameters of tagged and non-tagged (control) groups were compared by means of Student’s *t*-test. Analyses were performed using the SigmaPlot Version 13 for Windows (Systat Software Inc., Chicago, IL, United States). Multivariate partial least-squares discriminant analysis (PLS-DA) of data on respiratory frequency and physical activity index of exercised sea bream in the 1–6 BL/s water speed range was conducted with the EZ-info software (Umetrics, Sweden). The quality of the PLS-DA model was evaluated by R2Y and Q2Y parameters, which indicated the fitness and prediction ability, respectively.

## Results

**FIGURE 4:**
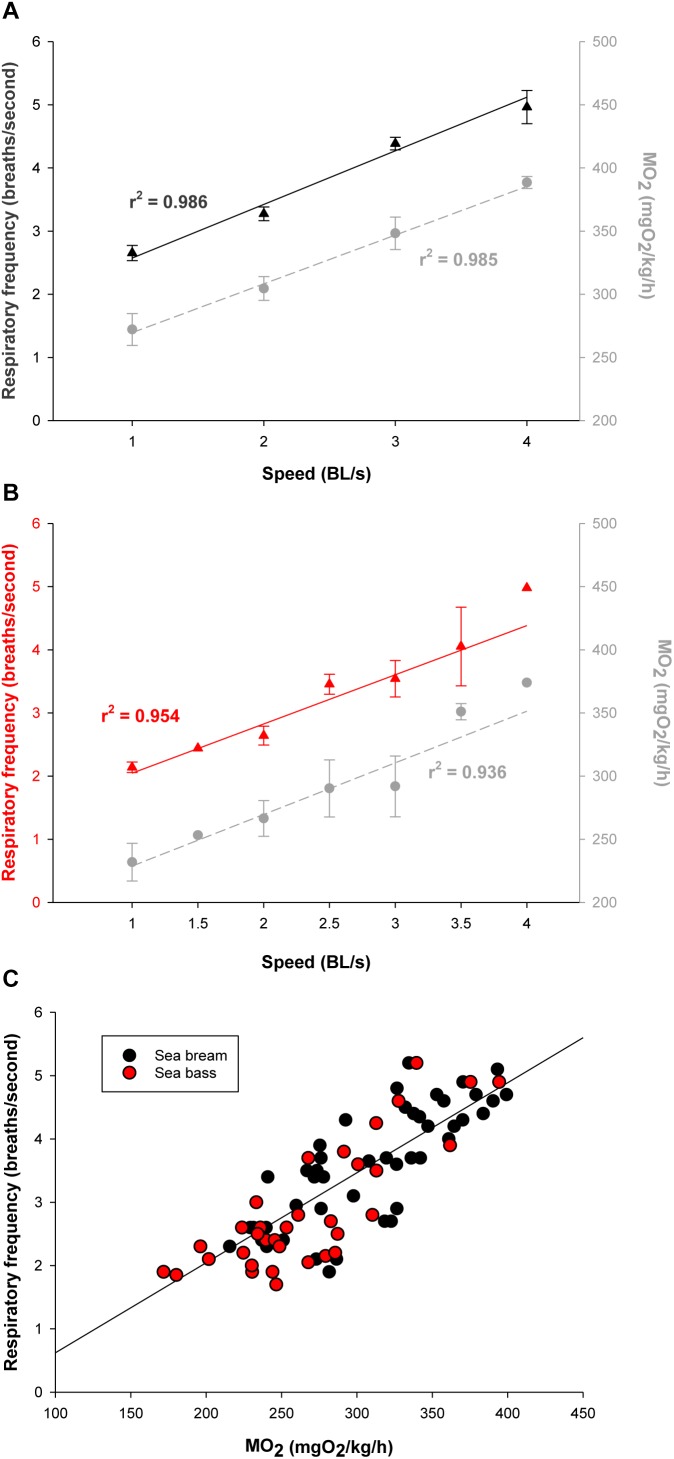
**(A)** Measurements of MO_2_ (mgO_2_/kg/h; gray circles) and respiratory frequency (breaths/s; black triangles) of sea bream (35–60 g body weight range) with increasing speed in the swim tunnel respirometer. Each point represents mean ± SEM of 6–10 measurements from a total of 16 fish. **(B)** Measurements of MO_2_ (mgO_2_/kg/h; gray circles) and respiratory frequency (breaths/s; red triangles) of sea bass (90–150 g body weight range) with increasing speed in the swim tunnel respirometer. Each point represents mean ± SEM of 6 measurements. **(C)** Correlation plot of MO_2_ and respiratory frequency values for each sea bream (black circles) and sea bass (red circles) individual.

**FIGURE 5:**
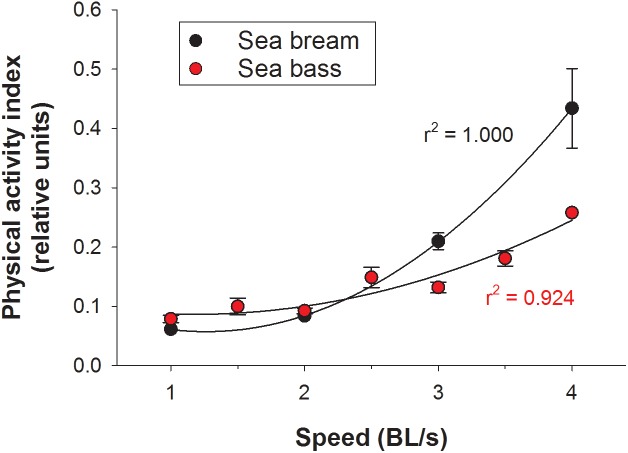
Output of physical activity index with increasing speed in the swim tunnel respirometer for sea bream (black circles) and sea bass (red circles). Each point represents mean ± SEM of 6–10 measurements.

Initials tests for the assessment of the functional significance of calculated outputs relied on PC post-processing of raw data. Challenges in the swim tunnel respirometer showed for both sea bream (Figure 4A) and sea bass (Figure 4B) a linear increase of MO_2_ in the 1–4 BL/s speed range. The calculated respiratory frequency by data post-processing paralleled MO_2_. At the same swimming speed, MO_2_ and the calculated respiratory frequency were consistently higher in sea bream than in sea bass. However, for a given MO_2_, the respiratory frequency was similar for both species as shown by the correlation plot of these two variables when all data were put together (Figure 4C). Regarding physical activity index, it increased exponentially rather than linearly with the increase of swimming speed, as the total jerk magnitude only reflects fish-related accelerations (Figure 5). This pattern was observed both in sea bream and sea bass, though it became more evident in sea bream at swimming speeds over 3 BL/s.

**FIGURE 6:**
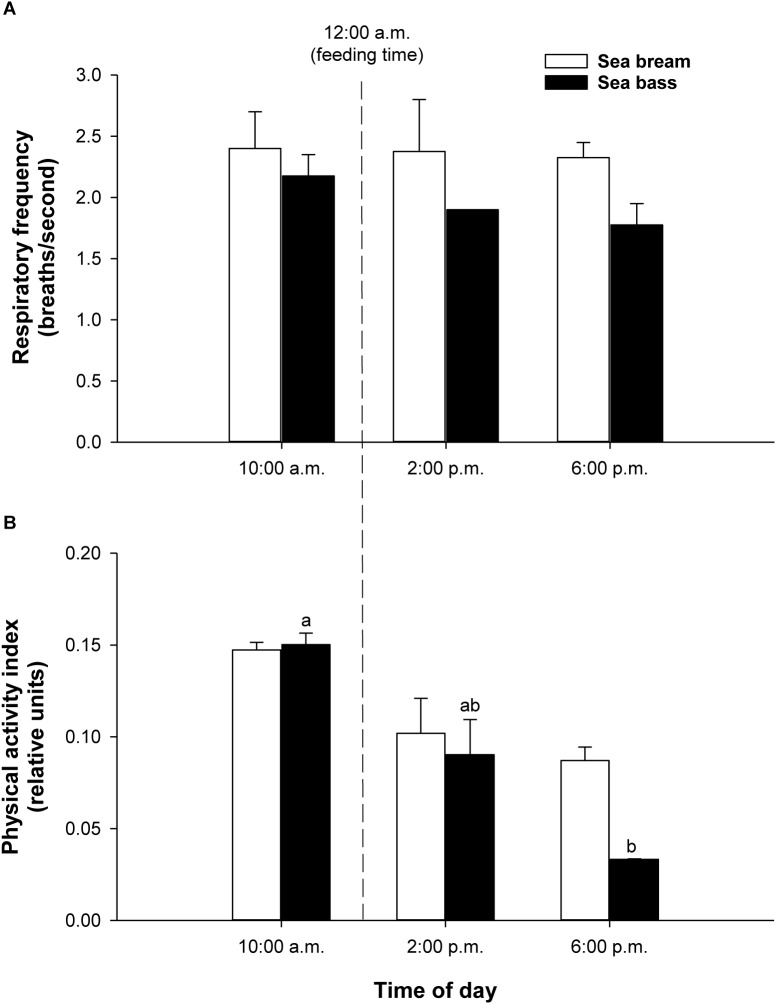
**(A)** Daily variation of respiratory frequency values in free-swimming sea bream (292.5 ± 20.5 g mean body weight; white bars) and sea bass (161.6 ± 14.9 g mean body weight; black bars) in 500-L tanks. Each bar represents mean ± SEM of 6 measurements. **(B)** Daily variation of physical activity values in free-swimming sea bream (white bars) and sea bass (black bars) in 500-L tanks. Each bar represents mean ± SEM of 6 measurements from the same 6 individuals. Different superscript letters indicate significant differences (*P* < 0.05; one-way ANOVA).

Registered values in free-swimming fish were in the measurable range by the PC-tested algorithms in the swim tunnel. In both fish species, the calculated respiratory frequency in holding tanks did not show significant daily variations from 10:00 a.m. to 6:00 p.m., ranging the respiratory frequency between 2.4–2.3 breaths/second in sea bream and 2.2–1.8 breaths/second in sea bass (Figure 6A). For the measurements of physical activity, the range of variation was also higher in sea bass, being statistically significant the decrease of physical activity from 0.150 ± 0.004 to 0.033 ± 0.001 relative units (*P* < 0.05). The observed decrease in sea bream varied from 0.147 ± 0.003 to 0.087 ± 0.005 relative units (*P* = 0.12) (Figure 6B).

**FIGURE 7:**
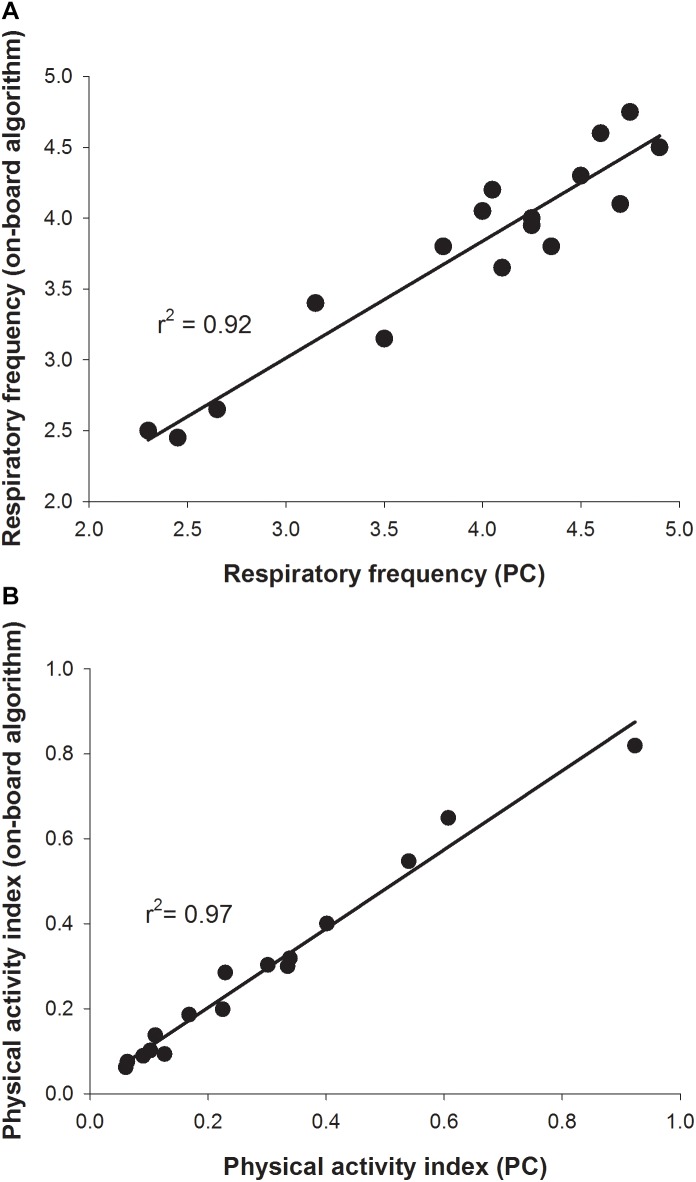
Validation of on-board algorithms from low to submaximal exercise. Values are mean of six sea bream juveniles (141.3 ± 7.4 g mean body weight). **(A)** Correlation plot for a given swimming speed between respiratory frequency values calculated for 2 min raw data post-processed on a PC (*x*-axis) or on-board (*y*-axis). **(B)** Correlation plot for a given swimming speed between physical activity index values calculated for 2 min raw data post-processed on a PC (*x*-axis) or on-board (*y*-axis).

**FIGURE 8:**
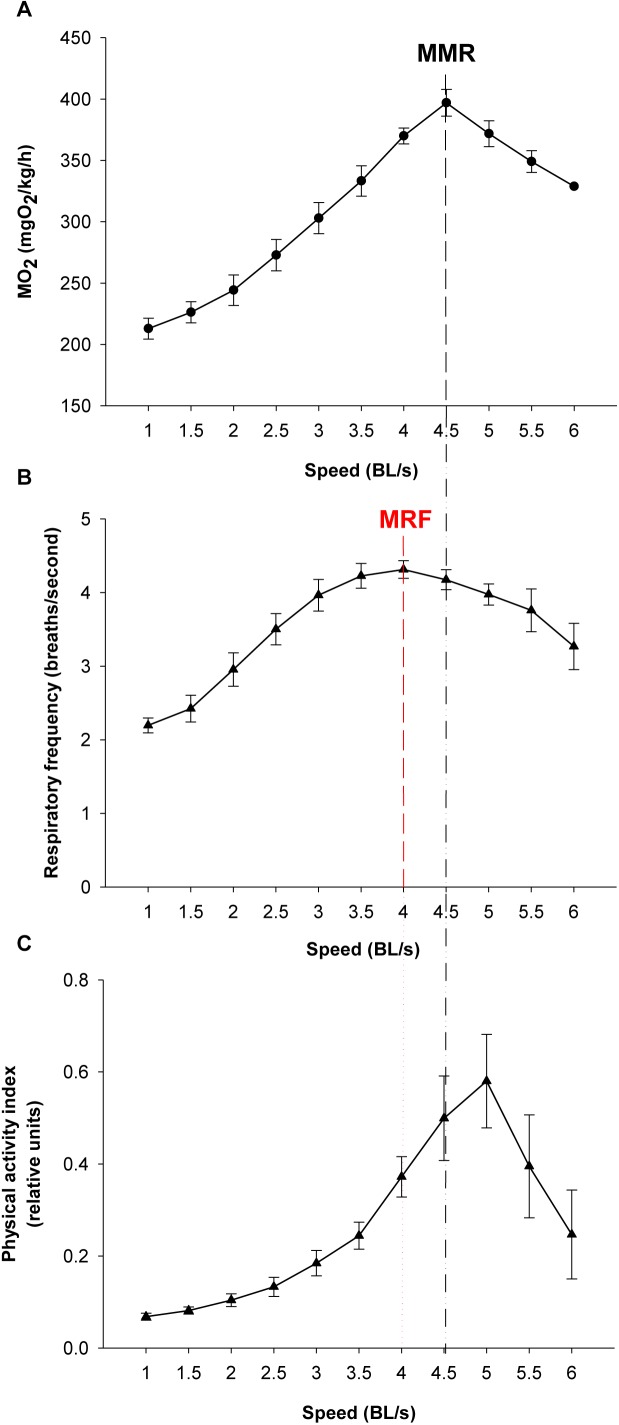
Respirometer and on-board output from low to submaximal exercise. Values are the mean ± SEM of six sea bream juveniles (95.2 ± 3.7 g mean body weight) **(A)** Respirometer measurements of MO_2_ (mgO_2_/kg/h) with increasing swimming speed. Maximum metabolic rate (MMR) is marked. **(B)** Respiratory frequency (breaths/s) with increasing swimming speed. The maximum respiratory frequency (MRF) is marked. **(C)** Physical activity index with increasing swimming speed.

**FIGURE 9:**
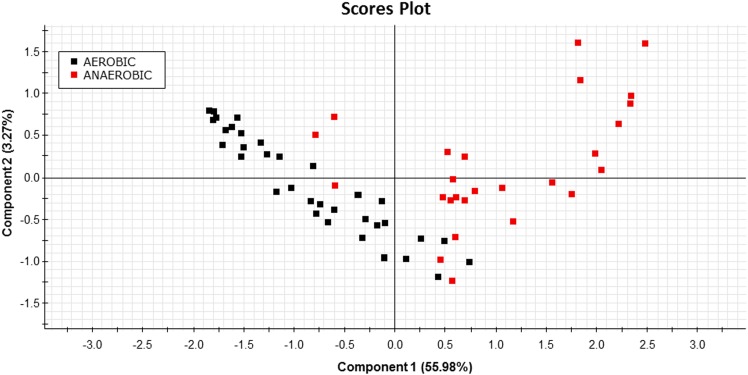
PLS-DA score plot of on-board calculated parameters at different swimming speeds before (black squares) and after (red squares) the achievement of the MMR in sea bream juveniles from low to submaximal exercise.

For validation of on-board algorithms, a second step of swim tunnel tests was conducted. Sea bream juveniles of 80–100 g were exercised over 1–6 BL/s until exhaustion (fish were considered exhausted when they rested at the back grid for at least 5 s). Simultaneous raw data storage and algorithm on-board calculations allowed the comparison of both approaches for the calculation of respiratory frequency and physical activity indexes over the same 2 min period, and close linear correlations near to 1 between on-board and PC-calculated values were found (Figure 7). The different dynamics of on-board calculated parameters from low to submaximal exercise were observed. Respirometer measurements of MO_2_ increased linearly up to a swim speed of 4.5 BL/s, paralleling the increase in the respiratory frequency, with a maximum respiratory frequency (MRF) close to 4 BL/s (Figure 8A,B). This short delay in the achievement of the maximum metabolic rate (MMR), defined as the maximum O_2_ consumption in exercised fish, might be due, at least in part, to the instantaneous nature of sensor measurements (gill breathing) compared to the buffered changes in the measurements of O_2_ concentrations in the 10-L chamber of respirometer. In any case, these two types of respiration measurements decreased progressively and markedly with the increased contribution of anaerobic metabolism close to submaximal exercise. Likewise, measurements of physical activity achieved a maximum activity at 5 BL/s. This was preceded by a slight decrease of slope at 4.5 BL/s that became clearly negative with the enhancement of the unsustainable anaerobic metabolism close to submaximal exercise (Figure 8C). Any of the analyzed variables was informative enough to ascertain the aerobic/anaerobic scope when considered individually. However, given their different dynamic patterns in response to exercise, multivariate analyses with on-board processed data resulted in a fairly good differentiation of aerobic/anaerobic fish condition along the first component, with a 59% of total variance explained (R2Y) and 57% predicted (Q2Y) by the two components of the discriminant model (Figure 9).

**FIGURE 10:**
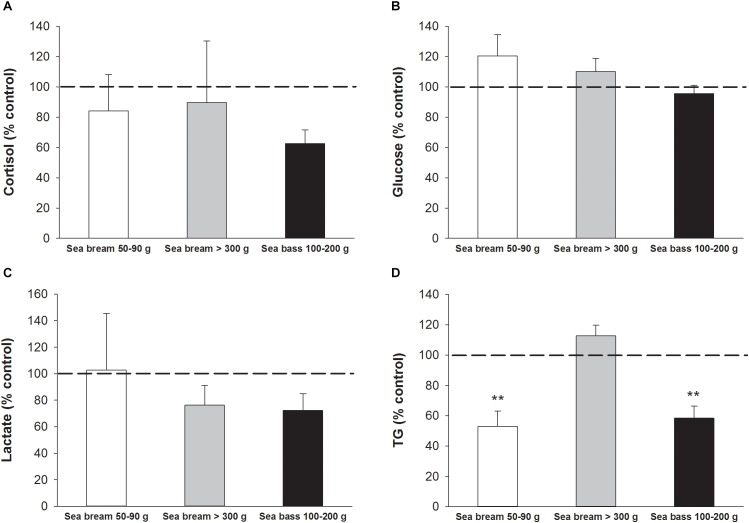
Plasma levels of **(A)** cortisol, **(B)** glucose, **(C)** lactate, and **(D)** triglycerides represented as a percentage of non-tagged control values. Each bar represents mean ± SEM of *n* = 5–7 for each fish species (sea bream, sea bass) and class of size (405.3 ± 13.9 g; 69.2 ± 2.6; 172.9 ± 14.9 g). Asterisks indicate statistically significant differences with control (*P* < 0.01, Student’s *t*-test).

The impact of device attachment in fish physiology was assessed by comparisons of circulating levels of markers of stress and welfare in tagged and non-tagged (control) free-swimming fish 1 week after implantation of dummy devices. No differences were found in circulating levels of cortisol (Figure 10A), glucose (Figure 10B) or lactate (Figure 10C) in 100–200 g sea bass nor sea bream for the two class of size analyzed. By contrast, TG levels in 50–90 g sea bream and 100–200 sea bass were significantly lowered (*P* < 0.01) in tagged fish in comparison with non-tagged ones (Figure 10D). This disturbance was not detected in larger fish (>300 g sea bream).

## Discussion

The present study is the proof of concept of the work conducted within the AQUAEXCEL^2020^ EU project for the design, programming and testing of a miniaturized device (AE-FishBIT v.1s) intended for individual fish phenotyping of metabolic condition and welfare. To achieve this end, different technological solutions already available as research tools or commercial products were initially considered (Tamayo et al., 2013; Bandodkar and Wang, 2014; Tamsin, 2015), but given the cost and methodological limitations when operating underwater, it was decided that the use of mechanical devices was more feasible than other options based on electric and/or optical sensors. The AE-FishBIT tri-axial accelerometer is able to register physical activity and operculum beats (two in one) as a direct measure of respiratory frequency. This sensor device was designed to work in stand-alone mode (no wireless data transmission) due to the constraints in size, weight, battery consumption and signal transmission in aquatic environments (Lloret et al., 2012; Climent et al., 2014). The AE-FishBIT also included a tag RFID system for an easier operability and data processing during fish data recording from resting to moderate or very active behavior under aerobic and/or anaerobic conditions.

When the system was tested in exercised juveniles, close correlations were found between O_2_ consumption and calculated respiratory frequency in both sea bream and sea bass. For measurements of physical activity, the jerk of accelerations increased exponentially rather that linearly with the increase of swimming speed (Figure 4, 5). Indeed, the jerk magnitude is independent of orientation and it only reflects accelerations, which are theoretically zero at the constant speeds imposed in the swim tunnel controlled conditions. It should then be assumed that when fish are induced to swim against the water current, the physical activity index will reflect the resistance of fish to match current water speed. Then, the steady increase of this index could be related with the number of intermittent (burst-and-coast) swimming bursts (Marras et al., 2010). Further increase in water speed should force a transition from burst-and-coast to continuous swimming that would decrease the number of burst and consequently decrease the physical activity index under forced swimming conditions (Figure 8C). Thus, the vigorous and irregular tail movements of fish resulted in a non-linear increase of the jerk of accelerations. This activity feature was more evident in the case of sea bream, which probably reflects a lower capability for fast swimming in comparison to sea bass with the spindle-shaped body characteristic of an active predator (Spitz et al., 2013), as indicates its common name in France “le loup de mer”. Likewise, for a given MO_2_, measurements of jerk accelerations were higher in free-swimming fish than in forced exercised fish (swim tunnel), where a more static position limits changes of trajectory and accelerations (Figure 4–6). From tests conducted across the light-phase in free-swimming animals, it is also conclusive that the range of variation was higher for measurements of physical activity rather than respiratory frequency, especially in the case of sea bass (Figure 6). However, this issue needs to be corroborated with a more continuous circadian data recording with the advent of new AE-FishBIT versions.

The usefulness of behavior monitoring remains yet to be fully exploited in aquaculture practice, though it is well known that swimming performance provides a complete measurement of animal fitness and perhaps stress behavior in fish kept under specific environmental conditions (Nelson, 1989; Plaut, 2001; Remen et al., 2016). Thus, measurements of MO_2_, and secondly respiratory frequency, can be considered a good measurement of the energy expended by fish to integrate a wide-range of physiological processes influenced by both endogenous (e.g., size, age, and fish strain) and exogenous (light, temperature, O_2_ concentration, time of day, etc.) factors. In our experimental conditions, MO_2_ increased in sea bream and sea bass from 230–270 to 370–400 mgO_2_/kg/h with the increase of swimming speed from 1 to 4 BL/s (Figure 4A,B). These reported values are in line with those found in previous studies in sea bream (Martos-Sitcha et al., 2018), European sea bass (Claireaux et al., 2006) or other farmed fish, such as meager (*Argyrosomus regius*) (Peixoto et al., 2017), challenged at water temperatures close to those set in our experimental approach. Thus, given the close parallelism between measurements of MO_2_ and respiratory frequency, we can conclude that the AE-FishBIT provided reliable results of O_2_ consumption in a wide-physiological range not only when the energy requirements did not exceed the O_2_ demand for a sustainable aerobic metabolism, but also during the anaerobic phase that was largely increased at submaximal exercise (Ejbye-Ernst et al., 2016). This was demonstrated by additional swimming tests (Figure 7–9), prolonged until fish exhaustion at 6 BL/s, which also served to corroborate the suitability of on-board algorithms of respiratory frequency and physical activity through aerobic and anaerobic conditions.

The weight of the first AE-FishBIT prototype is 600 mg in air with an estimated 50% of buoyancy. These features make it very convenient for its use in fish of 30–35 g onwards, according to the empirical “2% tag/body mass” rule for implanted devices (Winter, 1996; Jepsen et al., 2004). Recent works claim that this rule can be even extended for some fish species up to 7% of body mass without detrimental effects on performance or survival (Smircich and Kelly, 2014; Makiguchi and Kojima, 2017). In the present study, we assessed the influence of device implantation on fish welfare by measurements of blood biochemical parameters in both sea bream and sea bass, and no differences were found between tagged and non-tagged fish in classical stress markers (cortisol, glucose, and lactate) 1 week after device attachment regardless of body weight (50->300 g). During this time, the observed feeding behavior was also considered to be quite similar

in tagged and non-tagged fish. However, the observed decrease in plasma TG levels in tagged 100–200 g sea bass and 50–90 g sea bream would be indicative of, at least, a transient impairment of feed intake. This finding was not observed in the largest sea bream (>300 g), being this fact indicative of the importance of defining the critical fish size to minimize the impact of tag burden in welfare and behavior in different aquaculture scenarios (Figure 10). In parallel, important research efforts continue to be made to reduce the dimensions and weight of upcoming AE-FishBIT prototypes, keeping or even increasing the functional features of the device. At short-medium term, the main envisaged hardware strategies for this miniaturization will consider the use of: (i) flexible kapton instead of the current pcb rigid substrate, (ii) lighter and smaller long-life batteries and (iii) improved circuit packaging architecture based on Flip Chip (Lau, 2016) or embedded wafer level ball grid array (eWLB) technologies (Meyer et al., 2008). These solutions are based on currently available commercial components, but as a part of semi-industrialization packaging and production we cannot exclude the design of custom integrated circuits as the most ambitious and technologically challenging procedure for the final product miniaturization.

Data processing and software improvements with on-board algorithms are also key steps on the prototype development due to its major impact on data recording autonomy, which makes possible different short- and long-time schedules, adjusted for instance to 2 days (2 min recording each 15 min), 8 days (2 min recording each 60 min) or 24 days (2 min recording each 180 min). This relatively high autonomy also requires a low computational load, which did not compromise the usefulness of the microprocessor mathematical approximations, as demonstrated by the correlations of PC and on-board outputs in sea bream juveniles, exercised from low to submaximal exercise (Figure 7). This long coverage of exercise activity also highlighted that the system remained highly informative of metabolic condition with the shift of aerobic to unsustainable anaerobic metabolism, which reduces the efficiency of ATP production and promotes the accumulation of deleterious by-products (e.g., H^+^, lactate) (Richards, 2009; Seibel, 2011). Indeed, aerobic locomotor activity is powered by red oxidative muscle fibers, but when approaching their maximum power capacity, a gait transition to anaerobic fueling is assisted by the activation of fast white muscle fibers that results in fatigue and depletion of muscle glycogen depots (Kieffer, 2000; Sänger and Stoiber, 2001). Some degree of activation of anaerobic metabolism occurs before reaching MMR, as it has been determined in different experimental models (Burgetz et al., 1998; Lee et al., 2003; Hinch et al., 2006; Teulier et al., 2013), including sea bream and guppy (*Poecilia reticulata*) (Ejbye-Ernst et al., 2016). However, as pointed out before, the closely related MRF and MMR will mark the start of a burst-assisted swimming, resulting in a change of metabolic scope and swimming energy partitioning (Peake and Farrell, 2004; Peake, 2008; Marras et al., 2013; Svendsen et al., 2015). Importantly, this energy transitioning can be monitored by the AE-FishBIT device, as discriminant multivariate analysis distinguished two main groups of fish behavior when the achieved MRF-MMR was taken as main criteria of aerobic/anaerobic classification (Figure 9). This complex trade-off is mediated by O_2_ sensors (Norin and Clark, 2016), being defined the O_2_ limiting saturation (LOS) as the O_2_ threshold level that is no longer sufficient to maintain MO_2_ at a given temperature and voluntary swimming activity (Remen et al., 2013, 2015). Acute hypoxia drives to the re-adjustment of sea bream mitochondrial machinery at transcriptional level, leading to a more efficient O_2_-carrying capacity in blood cells (Martos-Sitcha et al., 2017). Different types of adaptive responses also occur under moderate hypoxia (above LOS), which reflects the tissue-specific responsiveness of liver, heart, skeletal muscle and blood cells, according to their metabolic capabilities and O_2_ availability (Martos-Sitcha et al., unpublished). Indeed, fish transcriptomic meta-analysis in response to a vast array of challenged conditions highlights the key role of mitochondria in front of different cellular stresses, including hypoxia, hypercortisolism and malnutrition (Calduch-Giner et al., 2014). In particular, sea bream juveniles exposed to thermal stress or multiple sensory perception stressors (shaking, sound, light flashes, and water flow reversal) show adaptive responses of glycolytic pathways and mitochondrial respiratory chain (Bermejo-Nogales et al., 2014). How these adjustments at cellular level can be correlated with the monitored AE-FishBIT parameters is the upcoming envisaged challenge in a scenario of global change with increases of temperature, ocean acidification and reduced O_2_ concentrations (Gruber, 2011).

Another important challenge is the improvement of the device attachment procedure to the operculum. External tagging procedures in fish are usually related to telemetry devices in large fish, with application near the dorsal or anal fins (Jepsen et al., 2015). At the present stage, the RapID tag and 3D pocket approach has served for the short-term validation experiences, but this system procedure was not operative for more than 1–2 weeks due to the appearance of macroscopic signals of damage and necrosis caused by loosening at the piercing location. This problem has been currently solved with the use of corrosion-resistant self-piercing fish tags (National Band & Tag Company, Newport, KY, United States) as the AE-FishBIT support. In our hands, the complete attachment implantation procedure takes less than 1 min, but automatization procedures like those used for massive fish vaccination (Skala Maskon, Stjørdal, Norway) need to be implemented for a more practical and routine use of this device in fish farming. In any case, specific attachment procedures need to be validated for each fish species, size and physiological condition in different aquaculture scenarios through production cycles.

**FIGURE 11:**
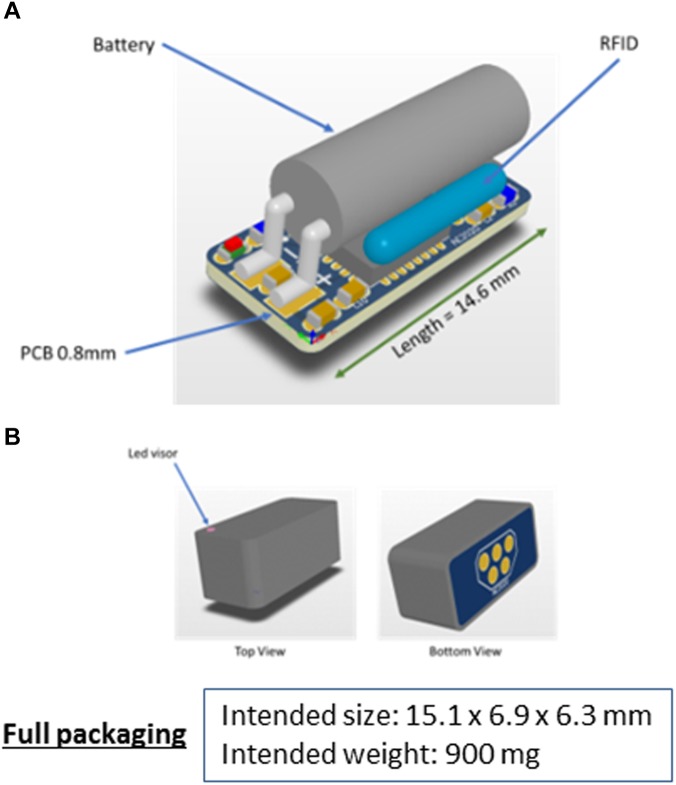
Schematic 3D view of the intended AE-FishBIT v.2 prototype before **(A)** and after packaging **(B)**.

Further improvements envisaged in the AE-FishBIT device include a more compact design (Figure 11) with the reallocation of the connection pins for charge and data transmission in the bottom side of pcb. The enclosure of the device is a protective packaging that completely covers the edge of the printed circuit, which will improve the insulation of the sensor components. Additionally, the new printed circuit is manufactured in multilayer technology without through holes. The combination of the compact pcb and the protective enclosure results in a fully sealed device with a minimum increase of the total weight (around 900 mg). The design can stand water contact with the connector pins and the supply voltage of the battery is protected by a diode that prevents its discharge. During the experiments, data transmission signals are always in the high impedance state, which prevents the leakage of electrical current. The new intended prototype (AE-FishBIT v.2) has been tested underwater for more than 24 h without any additional protection in the electrical contacts. For longer experimental periods, the electrical contacts will be further protected with an adhesive waterproof lid. The removable cap will be adapted to be combined with the best attachment procedure for each fish species and experiment protocols.

## Conclusion

A miniaturized device to register at the same time fish operculum beats (respiratory frequency) and physical activity has been designed, produced and tested in sea bream and sea bass as the proof of concept of the functional significance of the calculated data. The basic operating mode is stand-alone with autonomy of 6 h of continuous data recording with different programmable schedules. Validity and functional significance of tri-axial accelerations has been assessed under forced and voluntary exercise, and further work is underway to improve semi-industrial packaging and production. From a functional point of view, data analysis depicts the potential use of the proposed prototype for assessing the gait transition of metabolic scope and energy partitioning. The expected impact is the improvement of metabolic phenotyping in a slight invasive manner for the implementation of fish management and selective breeding. The prototype can also contribute to establish more strict and reliable welfare standards, and a better perception of quality controls in aquaculture production.

The prototype is protected by a registered patent (P201830305).

## Ethics Statement

All procedures were approved by the Ethics and Animal Welfare Committee of Institute of Aquaculture Torre de la Sal and carried out according to the National (Royal Decree RD53/2013) and the current EU legislation (2010/63/EU) on the handling of experimental fish.

## Author Contributions

JP-S coordinated the different research teams. JS, DR-V and JM-N assembled the device components and implemented software programming. CC-D and MF established mathematical parameters for data outputs and on-board algorithm approximations. FB, EC, AV, and ML worked on device packaging and insulation procedures. JP-S, JM-S, and JA conducted functional tests. JM-S, JC-G, and JP-S wrote the manuscript. All authors edited the manuscript and read and approved the final manuscript.

## Conflict of Interest Statement

The authors declare that the research was conducted in the absence of any commercial or financial relationships that could be construed as a potential conflict of interest.

## Footnotes

1 www.enviguard.net
